# Update on Practical Management of Early-Stage Non-Small Cell Lung Cancer (NSCLC): A Report from the Ontario Forum

**DOI:** 10.3390/curroncol31110514

**Published:** 2024-11-08

**Authors:** Parneet K. Cheema, Paul F. Wheatley-Price, Matthew J. Cecchini, Peter M. Ellis, Alexander V. Louie, Sara Moore, Brandon S. Sheffield, Jonathan D. Spicer, Patrick James Villeneuve, Natasha B. Leighl

**Affiliations:** 1 Division of Medical Oncology, William Osler Health System, Brampton, ON L6R 3J7, Canada; 2 Faculty of Medicine, University of Toronto, Toronto, ON M5S 1A8, Canada; 3 Department of Medicine, The Ottawa Hospital Research Institute, The Ottawa Hospital, University of Ottawa, Ottawa, ON K1H 8L6, Canada; pwheatleyprice@toh.ca (P.F.W.-P.); smoore@toh.ca (S.M.); 4 Department of Pathology and Laboratory Medicine, Western University, London, ON N6A 3K7, Canada; matthew.cecchini@lhsc.on.ca; 5 Department of Oncology, Juravinski Cancer Centre, McMaster University, Hamilton, ON L8V 5C2, Canada; ellisp@hhsc.ca; 6 Department of Medicine, Sunnybrook Odette Cancer Centre, Toronto, ON M4N 3M5, Canada; alexander.louie@sunnybrook.ca; 7 Department of Laboratory Medicine, William Osler Health System, Brampton, ON L6R 3J7, Canada; brandon.sheffield@williamoslerhs.ca; 8 Department of Thoracic Surgery, McGill University Health Centre, Montreal, QC H3G 1A4, Canada; jonathan.spicer@mcgill.ca; 9 Division of Thoracic Surgery, The Ottawa Hospital, University of Ottawa, Ottawa, ON K1H 8L6, Canada; pvilleneuve@toh.ca; 10 Department of Medicine, Princess Margaret Cancer Centre, University Health Network, University of Toronto, Toronto, ON M5S 1A8, Canada; natasha.leighl@uhn.ca

**Keywords:** early-stage non-small cell lung cancer (NSCLC), neoadjuvant NSCLC, adjuvant NSCLC, programmed death ligand 1 (PD-L1), epidermal growth factor receptor (EGFR), anaplastic lymphoma kinase (ALK)

## Abstract

Therapeutic strategies for early-stage non-small cell lung cancer (NSCLC) are advancing, with immune checkpoint inhibitors (ICIs) and targeted therapies making their way into neoadjuvant and adjuvant settings. With recent advances, there was a need for multidisciplinary lung cancer healthcare providers from across Ontario to convene and review recent data from practical and implementation standpoints. The focus was on the following questions: (1) To what extent do patient (e.g., history of smoking) and disease (e.g., histology, tumor burden, nodal involvement) characteristics influence treatment approaches? (2) What are the surgical considerations in early-stage NSCLC? (3) What is the role of radiation therapy in the context of recent evidence? (4) What is the impact of biomarker testing on treatment planning? Ongoing challenges, treatment gaps, outstanding questions, and controversies with the data were assessed through a pre-meeting survey, interactive cases, and polling questions. By reviewing practice patterns across Ontario cancer centers in the context of evolving clinical data, Health Canada indications, and provincial (Cancer Care Ontario [CCO]) funding approvals, physicians treating lung cancer voiced their opinions on how new approaches should be integrated into provincial treatment algorithms. This report summarizes the forum outcomes, including pre-meeting survey and polling question results, as well as agreements on treatment approaches based on specific patient scenarios.

## 1. Introduction

Non-small cell lung cancer (NSCLC) remains the most commonly diagnosed cancer and one of the leading causes of cancer-related mortality in Canada and worldwide [1,2,3,4]. Due to the propensity for distant metastases and early relapse, even patients diagnosed with early-stage disease have poor outcomes [5]. The 5-year overall survival (OS) is 60% for localized disease (stages I and II without nodal involvement) and 33% for locally advanced disease—indicating an urgent need for better systemic disease control [6,7].

Although there have been modest improvements in therapeutic strategies for early-stage NSCLC over the last two decades, strategies for metastatic NSCLC have evolved with the use of therapies directed against mutant oncoproteins and immune checkpoint inhibitors (ICIs; programmed cell death protein 1 [PD-1] and its ligand [PD-L1]) that promote host antitumor response. It is only recently that the clinical development of ICIs has shifted to early-stage NSCLC neoadjuvant and adjuvant settings and demonstrated remarkable efficacy with improved clinical outcomes. Table 1 summarizes recent trials in early-stage NSCLC.

**Figure 1:**
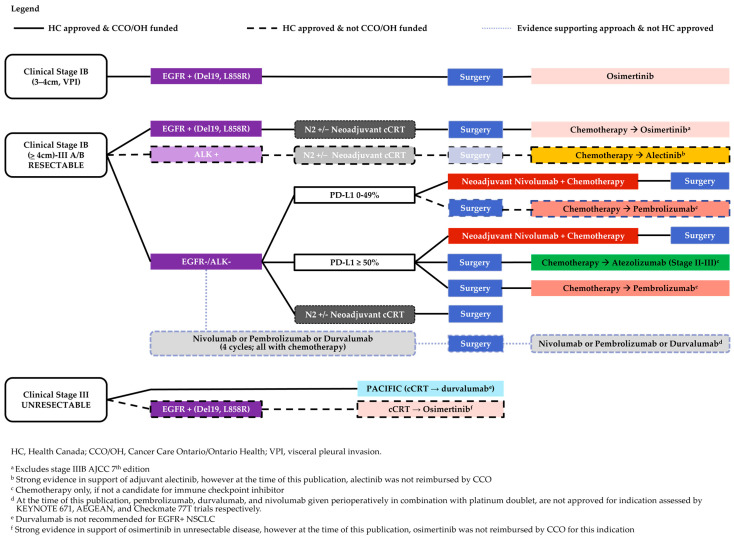
Curative-intent strategies based on Current Health Canada Approvals and CCO/OH funding.

Based on positive phase 3 trial results, Health Canada has approved atezolizumab, pembrolizumab, osimertinib, and alectinib as adjuvant treatments and nivolumab with chemotherapy as a neoadjuvant treatment option in adult patients with resectable NSCLC (Figure 1). Yet, there are questions on the magnitude of benefit associated with these approaches and how to best tailor them to disease stage, biomarker expression, and other patient-specific factors.

Considering the rapid data readouts in the context of slow reimbursement and funding approval processes in Canada and the lack of guidance on how to incorporate recent evidence in routine practice, Ontario physicians treating lung cancer perceived a need for a multidisciplinary forum to navigate a path forward in this therapeutic space.

This report provides a summary of key takeaways from the meeting. It highlights the outcomes of recent clinical trials in the context of the multimodality and multidisciplinary management of early-stage NSCLC in Ontario. Incorporating systemic therapies into neoadjuvant, adjuvant, or perioperative settings presents a significant shift in the early-stage NSCLC treatment paradigm and highlights the increasing need to discuss patient management within a multidisciplinary cancer conference (MCC). With several newly approved neoadjuvant and adjuvant approaches, collaboration between surgeons, pathologists, and medical and radiation oncologists will become increasingly relevant for decision-making. Surgeons and pathologists must be made aware not only of various systemic options but also of the funding requirements and access-related challenges (e.g., how long it takes to access the treatment, molecular testing requirements, the paperwork required, etc.) so that patients are referred to medical oncologists and their cases are discussed at MCCs in a timely manner. Furthermore, comprehensive and transparent discussions with patients, including their preferences on proposed treatment strategies, should be an integral component of the treatment plan.

## 2. Methods

The Ontario Forum for the Management of Early-stage NSCLC was convened as a means for Ontario physicians treating lung cancer to further discuss the impact and the adoption of recent clinical evidence in routine practice. The hybrid meeting took place on 28 September 2023, and medical oncologists, pathologists, radiation oncologists, and surgeons involved in managing lung cancer in Ontario were invited. Seventy-seven physicians from 23 oncology centers, representing all 14 provincial cancer programs, attended the meeting. The objectives of the meeting were to: 1. Review and evaluate the impact of the latest practice-changing data for neoadjuvant, adjuvant, and consolidation treatments in patients with early-stage NSCLC; 2. describe the current state of clinical practice for the treatment of patients with early-stage NSCLC across Ontario; and 3. summarize optimal and acceptable treatment practices in Ontario for early-stage NSCLC to steer a path forward in this therapeutic space through discussion of patient cases and clinical scenarios.

### 2.1. Pre-Meeting Survey

Prior to the meeting, a survey was sent to 23 cancer centers that participated in the forum, and representatives from 20 centers responded. The questions asked participants to rank treatment options for different stages of the disease, taking into consideration histology (squamous vs. non-squamous), smoking status, PD-L1 expression (0% vs. 1–49% vs. ≥50%), tumor size, and nodal involvement. For each question, a rank score was calculated per treatment (the highest rank score indicating the most preferred treatment option):

(1) $$\begin{array}{l} \mathrm{Rank}\;\mathrm{score}\;\mathrm{sum}=[(\mathrm{first}\;\mathrm{choice}\;n\times 8)+(\mathrm{second}\;\mathrm{choice}\;n\times 7)+(\mathrm{third}\;\mathrm{choice}\;n\times 6)+(\mathrm{fourth}\;\mathrm{choice}\;n\times 5)+(\mathrm{fifth}\;\\ \mathrm{choice}\;n\times 4)+(\mathrm{sixth}\;\mathrm{choice}\;n\times 3)+(\mathrm{seventh}\;\mathrm{choice}\;n\times 2)+(\mathrm{eighth}\;\mathrm{choice}\;n\times 1)] \end{array}$$

**Figure A1:**
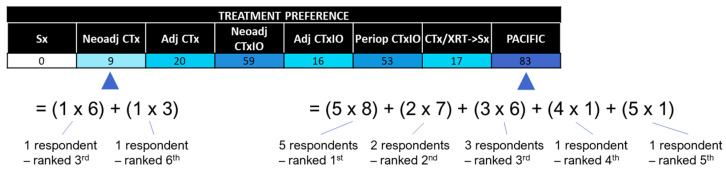
Example of a rank score calculation.

See an example of a rank score calculation in Appendix A (Figure A1).

### 2.2. Evidence Review

Recent large multicenter international phase 3 trials in adjuvant and neoadjuvant settings (Table 1) likely to impact the Canadian practice were identified, reviewed, and used for case-based discussions. The recent data were examined from the medical oncologists,’ surgeons’, radiation oncologists’, and pathologists’ perspectives.

### 2.3. Interactive Case Discussion

Three patient cases were developed to enable further discussion and understanding of clinician preference for a specific therapeutic approach. During the meeting, polling questions were used to assess preferred treatment options based on initial presentation, patient performance status, smoking history, tumor size, histology, disease stage, and nodal involvement. Each case included several what-if scenarios to assess whether and how different aspects of disease and/or patient characteristics impact treatment decisions.

## 3. Results

### 3.1. Pre-Meeting Survey

**Figure A2:**
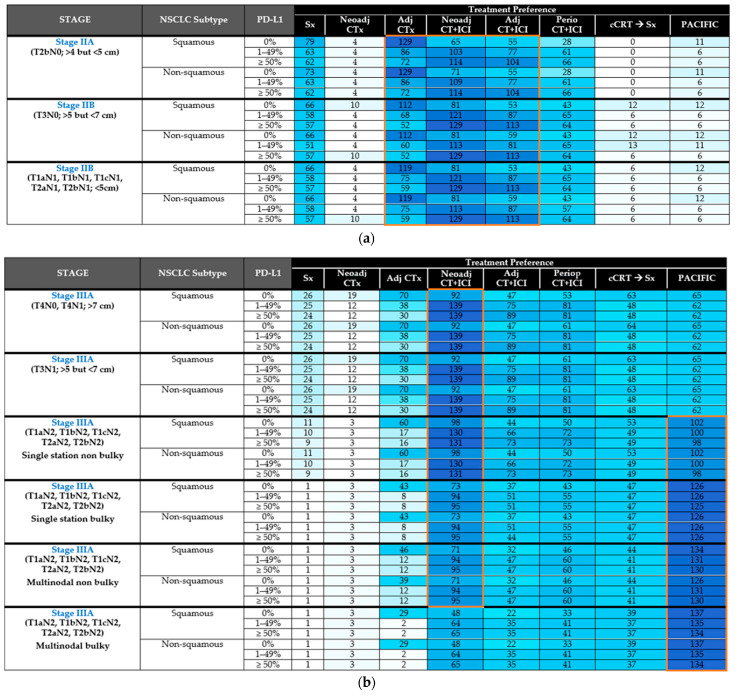

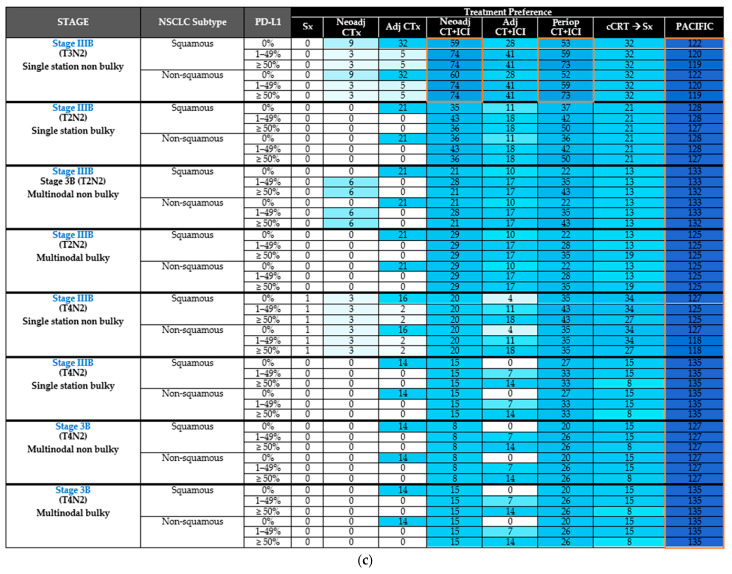
Pre-meeting survey treatment preferences based on disease stage, histology, and PD-L1 expression: (**a**) stage IIA and IIB; (**b**) stage IIIA; (**c**) stage IIIB. Sx, surgery; CT, chemotherapy; ICI, immune checkpoint inhibitor; cCRT, concurrent chemoradiation therapy.

The questionnaire revealed an increase in the preference for systemic therapy, particularly neoadjuvant chemotherapy + ICI, with an increase in disease stage (Table 2; Appendix B, Figure A2).

The preference for an ICI, especially in the neoadjuvant setting, increased with increased PD-L1 expression. 15 of 20 respondents noted no difference in treatment preferences for non-smokers at any stage of the disease or PD-L1 status. For patients with Stage IIIA, N1 disease, a surgical approach was preferred over concurrent chemoradiation therapy (cCRT) followed by consolidation with durvalumab (the PACIFIC trial protocol) [8]. Preference for the PACIFIC trial protocol increased by the size of the primary tumor and nodal involvement (size, location, and number of stations involved). According to the pre-meeting survey, the presence of actionable mutations beyond EGFR, ALK, and ROS1 alterations does not seem to affect therapeutic decisions. However, it became apparent in the case discussion that some actionable mutations could alter treatment decisions.

### 3.2. Supporting Evidence

When assessing the results of the recent trials, it is important to keep in mind that these trials used different American Joint Committee on Cancer (AJCC) staging criteria, which is also reflected in funding and access-related decisions.

Several trials assessed the efficacy of adjuvant targeted therapy (osimertinib in EGFR-positive [ADAURA] [11] and alectinib in ALK-positive [ALINA] [13]) or ICI (atezolizumab [Impower010] [14] and pembrolizumab [KEYNOTE-091] [16]) in stage IB-IIIA NSCLC. In the IMpower010 trial, the difference in OS between atezolizumab and best supportive care (BSC) was more pronounced in patients without EGFR/ALK mutations and with PD-L1 tumor cells ≥ 50% (5-year OS 89.1% with atezolizumab and 77.5% with BSC [HR 0.42 (0.23–0.78)] [15]. Based on this cohort of patients, Health Canada and, subsequently, CCO have restricted approval of adjuvant atezolizumab for patients with resected Stage II-IIIA that are PDL1 ≥ 50% and do not have EGFR mutations or ALK rearrangements. Subgroup analyses of the KEYNOTE-091 trial indicated that patients receiving adjuvant chemotherapy, even 1–2 cycles, had the greatest benefit from adjuvant pembrolizumab (HR 0.73 [95% CI 0.60–0.89]) compared to those who did not receive adjuvant chemotherapy (HR 1.25 [95% CI 0.76–2.05]) [23]. Thus, Health Canada restricted the approval of pembrolizumab to patients who had prior adjuvant chemotherapy. Interestingly, in the KEYNOTE-091 trial, there was a limited correlation between PDL1 status and the degree of improvement in disease-free survival (DFS) with pembrolizumab. Thus, Health Canada did not restrict the indication by PDL1 status.

Based on the CheckMate 816 trial [17], Health Canada and CCO approved nivolumab in combination with platinum doublet chemotherapy for the neoadjuvant treatment of adult patients with resectable NSCLC. A lower rate of distant recurrence events was reported in the nivolumab arm (10% vs. 22%), including a lower rate of central nervous system (CNS) recurrence (4% vs. 13%). However, the percentage of local recurrence events was similar in both arms (19% vs. 22%), indicating that most of the benefit of neoadjuvant therapy is in reducing the risk of distant recurrence and that more work is needed to mitigate locoregional recurrence (i.e., better surgeries, more extensive node dissection, and more attention to achieve R0 margins).

Perioperative strategies have been assessed in several phase 3 trials (pembrolizumab [KEYNOTE-671] [18], nivolumab [CheckMate 77T] [19], durvalumab [AEGEAN], toripalimab [Neotorch] [20], and tislelizumab [RATIONALE-315] [22]); however, none of the approaches have been Health Canada approved. Of relevance is that many participants in these trials did not undergo planned resection (7–18%) or did not have R0 margins after resection (5–17%). Disease progression as a reason for not undergoing surgery was cited in only 7–8% of patients and further illustrates the importance of looking at other factors to determine whether upfront surgery or cCRT followed by durvalumab is the best option.

The phase 3 PACIFIC trial [8] established consolidation durvalumab as the standard of care for patients with unresectable, stage III NSCLC with no disease progression following cCRT. However, as the trial did not demonstrate the benefits of durvalumab in EGFR/ALK-positive NSCLC, the use of consolidation durvalumab after curative-intent cCRT in patients with these alterations is not recommended. The phase 3 LAURA trial demonstrated clinically meaningful improvements with osimertinib in unresectable stage III EGFR-mutated NSCLC following definitive cCRT [10]. Several other trials are also addressing the role of targeted therapy as consolidation post definitive cCRT in patients with driver mutations, including ALK (HORIZON-01; NCT05170204) and RET (LIBERTTO 432; NCT04819100).

### 3.3. Insights Regarding Smoking Status, PD-L1 Expression, and Histology

#### 3.3.1. Smoking Status

Smoking status seems to impact the magnitude of benefits achieved with ICIs. Rates of pathologic complete response (pCR) in AEGEAN in non-smokers (3.9% with ICI and 0 with chemotherapy alone) and the rates of major pathologic response (MPR) in CheckMate 816 (10% with ICI and 5% with chemotherapy alone) as well as HRs for event-free survival (EFS) in KEYNOTE-671 (0.68 non-smokers vs. 0.52 smokers) and AEGEAN (0.76 non-smokers and 0.48 smokers) suggest more benefit with ICI in smokers vs. non-smokers.

#### 3.3.2. PD-L1 Expression

Although treatment with an ICI in neoadjuvant and perioperative settings had benefit irrespective of PDL1 expression levels, those with high PD-L1 expression (≥50%) seem to derive the most benefit in regard to response rates and EFS. Response rates in AEGEAN (pCR 9% in PD-L1 negative vs. 27.5% in patients with PD-L1 ≥50%) and CheckMate 816 (MPR: 29.5% in PD-L1 negative vs. 50% in PD-L1 ≥50%) as well as EFS in CheckMate 816 (HR for EFS 0.85 in PD-L1 negative vs. 0.24 in PD-L1 ≥50%), KEYNOTE-671 (HR 0.77 for PD-L1 negative vs. 0.42 for PD-L1 ≥ 50%) and AEGEAN (HR 0.76 for PD-L1 negative vs. 0.60 for PD-L1 ≥ 50%) were notably higher in patients with PD-L1 TPS ≥ 50%.

According to a meta-analysis [24], which included eight randomized clinical trials (CheckMate 816, KEYNOTE-671, NADIM II, AEGEAN, Neotorch, CheckMate 77T, TD-FOREKNOW, and RATIONALE 31), the pooled EFS estimate favored neoadjuvant ICI + chemotherapy over neoadjuvant chemotherapy alone (HR, 0.59; 95% CI, 0.52–0.67) regardless of PD-L1 expression. However, while there was an improvement in OS for patients treated with neoadjuvant ICI+ chemotherapy compared to neoadjuvant chemotherapy alone among patients with tumor PD-L1 levels of ≥1% (HR, 0.49; 95% CI, 0.33–0.73), this improvement was not observed for patients with tumor PD-L1 levels <1% (HR, 0.89; 95% CI, 0.66–1.19). This suggests that the degree of pre-operative benefit from ICI depends on the level of PD-L1 expression and that the risk/benefit ratio of ICI in patients with PD-L1 levels < 1% should be carefully evaluated when developing treatment plans, taking into consideration PD-L1 expression along with other patient- and disease-related characteristics.

#### 3.3.3. Histology

Histology does not seem to impact overall outcomes, although pCR rates in AEGEAN were lower in patients with adenocarcinoma vs. squamous cell carcinoma (13.3% vs. 23%). However, when interpreting these results, one should keep in mind that the trials were underpowered, and subgroup post-hoc analyses had a limited number of patients.

## 4. Clinical Dilemmas and Remaining Questions

To answer the questions of the forum, which was to determine to what extent patient (e.g., history of smoking) and disease (e.g., histology, tumor burden, nodal involvement, genomic alterations) characteristics influence treatment decisions, surgical consideration, and the role of radiation therapy, three cases were discussed.

**Figure 2:**
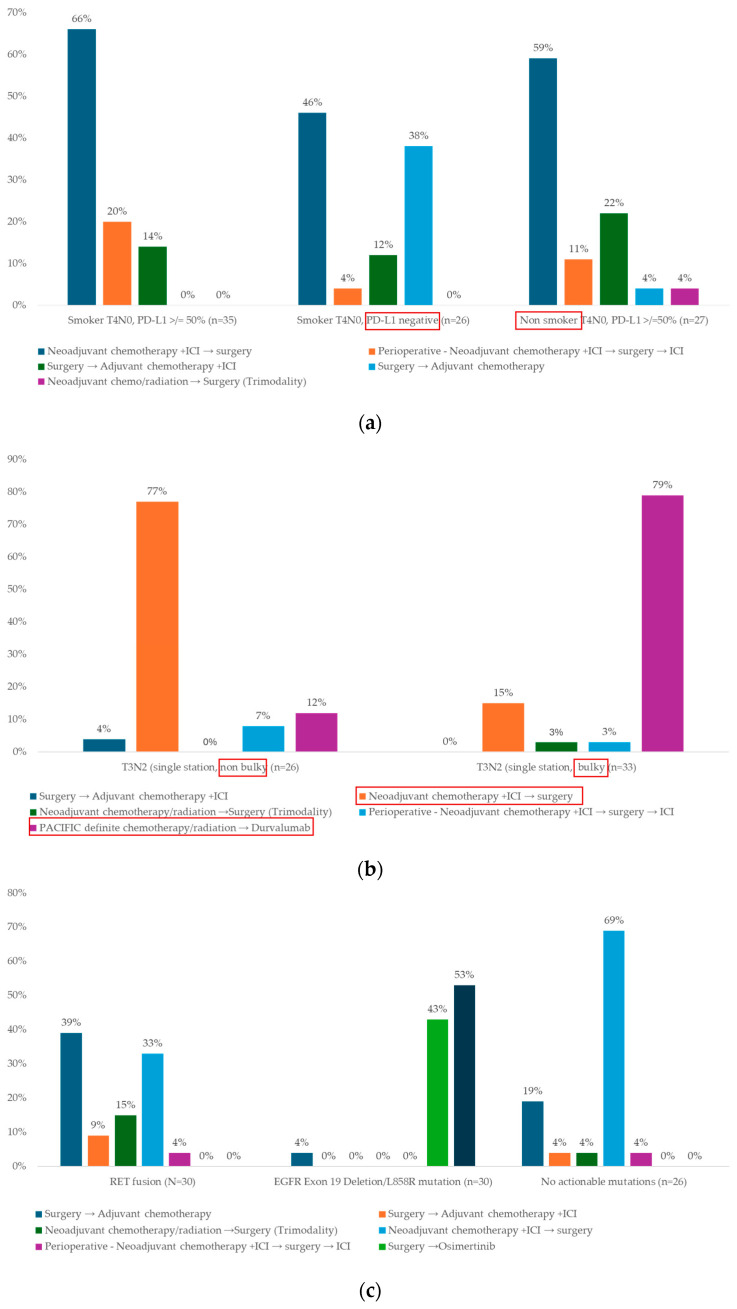
Preferred treatment approaches based on: (**a**) smoking history and PD-L1 expression, case #1; (**b**) nodal involvement, case #2; (**c**) genomic alterations, case #3. N indicates the number of participants that answered each question.

- Case #1 addressed disease histology, smoking history, PD-L1 expression, and heterogeneity of presentation of stage III.
- Case #2 examined how nodal involvement impacts treatment approaches.
- Case #3 assessed the impact of genomic alterations.
- Polling questions revealed differences in preferred treatment approaches based on smoking history and PD-L1 expression (Figure 2a), nodal involvement (Figure 2b), and genomic alterations (Figure 2c). While the preference for systemic therapy, particularly neoadjuvant chemotherapy + ICI, increased with the increase in disease stage, disease histology (squamous vs. non-squamous) did not impact treatment preference.

### 4.1. Case #1: The Impact of Disease Histology, Smoking History, PD-L1 Expression, and Heterogeneity of Presentation of Stage III

A 75-year-old man with a 35-pack-year history of smoking, ECOG PS 1, without autoimmune conditions or considerable comorbidities apart from hypertension and a history of superficial bladder cancer. Imaging studies reveal a 7.3 cm mass in the right lower lobe (RLL) of the lung, with no evidence of mediastinal adenopathy, brain metastases, lymphadenopathy, or distant metastases. Pathology from an RLL lung biopsy indicates TTF1 positive adenocarcinoma with a PD-L1 TPS of 60% and no actionable mutations identified through NGS. Endobronchial ultrasound (EBUS) confirms no nodal involvement, leading to a diagnosis of stage III T4N0 NSCLC. Clinical assessments show the patient has dyspnea (MCR 1), FEV1 of 102%, and DLCO of 87%. A surgical consultation deems the patient a good candidate for surgery.

#### Case #1 Discussion

Considering recent trial data, the forum participants agreed that smoking history and PD-L1 expression should be factored into therapeutic decisions. They determined that neoadjuvant ICI + chemotherapy followed by surgery is the most appropriate option for this patient, regardless of disease histology (Figure 2a). However, the patient’s age, comorbidities, and the risk of chemotherapy and ICI-related toxicities in both neoadjuvant and adjuvant settings must also be taken into account. Additionally, timely access to molecular testing is crucial in deciding whether a patient should proceed to surgery or be treated with neoadjuvant chemotherapy and ICI.

The panel considered clinical scenarios where involvement or proximity to the brachial plexus or spine could complicate surgery for a 75-year-old patient. They noted that choosing to initiate neoadjuvant chemotherapy + ICI followed by surgery would exclude the option of subsequent durvalumab if the patient, for any reason, could not undergo surgery, as durvalumab is not funded in Ontario for such cases. Additionally, all neoadjuvant and perioperative trials included patients who were resectable, not borderline resectable. Consequently, the forum members agreed on adopting the PACIFIC trial protocol because this case was deemed borderline resectable, which involves cCRT followed by durvalumab as consolidation therapy. For cCRT, the radiation dose would be determined based on the proximity to the spinal canal. Pain control is a significant concern and delivering the planned radiation doses may not always be feasible.

### 4.2. Case #2: The Impact of Nodal Involvement on Treatment Approaches: Surgical Considerations and the Role of Radiation Therapy

A 71-year-old man with chronic obstructive pulmonary disease (COPD) and mild chronic syndrome of inappropriate antidiuretic hormone secretion (SIADH), who is a lifelong smoker, initially presented with pneumonia, leading to the discovery of lung cancer. A biopsy of the right upper lobe (RUL) mass revealed squamous cell carcinoma with a PD-L1 TPS of 25%. PET scan results showed a T3N2 stage with a 5.8 cm RUL mass and involvement of a 16 mm non-bulky 4R lymph node. Surgical consultation indicated that his pulmonary function tests (PFTs) are adequate, and he is considered a candidate for surgical resection.

#### Case #2 Discussion

The optimal treatment for patients with N2 nodal disease remains controversial due to significant heterogeneity and the lack of a universally agreed-upon definition of resectability. This is particularly relevant in the context of the PACIFIC trial, which demonstrated good long-term outcomes with cCRT followed by consolidation with durvalumab without the need for a surgical approach. Preoperative surgical evaluation for these patients can be challenging and depends on various factors, including an individual surgeon’s expertise and the experience and capacity of the surgical center. The decision on resectability is subjective and based on a surgeon’s judgement of achieving R0 resection margins. Additionally, a tumor may be considered resectable but deemed medically inoperable due to the patient’s overall health, comorbidities, or high operative risk. Table 3 outlines some of the criteria for resectability and operability that could be considered.

Traditionally, patients with non-bulky nodal disease (one lymph node station involved, not bulky by size criteria of <3 cm) could undergo neoadjuvant cCRT followed by surgery or surgery followed by adjuvant chemotherapy. However, with the paradigm shift, the forum participants felt this patient could also receive neoadjuvant ICI + chemotherapy, as illustrated in Figure 2b. The decision regarding neoadjuvant ICI and chemotherapy should be left to the discretion of a medical oncologist, considering that comorbidities and performance status might preclude treatment with ICI. Therefore, it is crucial that surgeons do not over-promise and should advise patients regarding systemic treatment options only after review by a medical oncologist. Similarly, medical or radiation oncologists should not make commitments about the resectability of tumors or the medical operability of patients, deferring instead to thoracic surgeons.

If multiple nodal stations were involved and/or if nodes were bulky (i.e., ≥3 cm), the PACIFIC trial protocol with cCRT followed by durvalumab could be considered, as illustrated in Figure 2b. Should the surgical team deem the patient resectable, the operability and potential for neoadjuvant ICI + chemotherapy would be further discussed at an MCC. See Table 4 for key takeaways on resectability and the impact of nodal disease.

It is important to note the lack of data supporting post-surgical approaches in patients with positive resection margins or early locoregional relapses. However, the CheckMate 816 trial indicated that patients with positive resection margins benefited from neoadjuvant therapy. The 2-year EFS rate in patients with R1 (14% of patients) and R2 (19% of patients) margins was 62% [29]. Post-surgical treatments, including adjuvant radiation, likely contributed to EFS, suggesting that these patients require additional treatment approaches.

With the evolving use of neoadjuvant ICI therapy, the role of radiation in curative scenarios needs to be further defined. For patients who, after receiving neoadjuvant chemotherapy and ICI, are no longer candidates for surgical resection—either due to disease progression or other patient-related factors—the curative option would be to offer definitive radiation with or without chemotherapy. The unanswered question is whether the PACIFIC protocol, which includes the addition of durvalumab, would be applicable post-cCRT in patients already treated with neoadjuvant chemotherapy and ICI, as the benefit remains unknown. Radiation oncologists also face challenges related to patient fitness for radiation therapy and determining the appropriate areas to contour post-neoadjuvant therapy, specifically whether to target areas of previous disease or only the residual disease.

Chemoradiation therapy could also be considered for patients with large or locally invasive (T4) tumors with N2 disease, as well as those with superior sulcus tumors (SST). A multicenter, single-arm, confirmatory trial (CRES3T) investigated the efficacy and safety of three cycles of S-1 plus cisplatin combined with concurrent radical-dose thoracic radiotherapy (TRT; 66 Gy in 33 fractions), followed by surgery in patients with SST. SST was defined as a tumor that directly invades the chest wall, including the first rib or higher, the subclavian artery, or the subclavian vein [30]. Of the 60 enrolled patients, 49 underwent lobectomy and resection of the involved sites, achieving a complete resection rate of 98% (48/49). The 3-year OS and PFS rates were 73% (95% CI 60–83%) and 53% (95% CI 40–65%), respectively. The overall response rate and pCR rates were 42% (25/60, 95% CI 29–54%) and 33% (16/49, 95% CI: 20–46%), respectively. The CRES3T trial demonstrated that induction chemotherapy combined with concurrent radical-dose TRT followed by surgery was safe and effective in treating SST. Therefore, this strategy versus a neoadjuvant or perioperative ICI + chemotherapy approach will need to be individualized.

### 4.3. Case #3: The Impact of Genomic Alterations on Treatment Decisions

A 52-year-old man, a non-smoker with no significant comorbidities, presented with nonspecific abdominal pain that resolved on its own. During his evaluation, an incidental left lower lobe mass was discovered on a CT scan. Imaging revealed a 3.0 cm FDG-avid primary tumor in the left lower lobe with no lymph node involvement or distant metastases, and MRI confirmed no brain metastases. Pathology from EBUS indicated adenocarcinoma (TTF1 positive) with a RET fusion; PD-L1 was unknown. Pulmonary function tests showed FEV1 at 95% and DLCO at 71%, deeming him a good surgical candidate.

#### Case #3 Discussion

This case underscores the importance of rapid biomarker testing to determine the optimal treatment plan and demonstrates how treatment recommendations can shift based on biomarker status (Figure 2c). The use of ICIs in the neoadjuvant setting necessitates comprehensive biomarker analyses before making treatment decisions. Reflex testing, where the pathologist is responsible for initiating and managing testing for a predefined set of biomarkers, is crucial for this process [31]. However, despite being funded by Cancer Care Ontario for all patients with NSCLC [32], reflex biomarker testing in Ontario remains suboptimal.

While it might be appealing to use rapid single-gene assays (e.g., Idylla) in patients with early-stage disease to quickly assess EGFR, ALK, and PD-L1 expression within a few hours, this approach can have significant consequences. These include higher healthcare system costs and potential delays in treatment. Additionally, the identification of specific biomarkers is not only prognostic for the effectiveness of ICIs but also helps tailor treatment based on disease characteristics [33]. Relying on single-gene testing may also result in tissue exhaustion, which can complicate future biomarker analyses. Sheffield et al. evaluated the total costs of biomarker testing using NGS compared to single-gene testing strategies among newly diagnosed Canadian adults with metastatic NSCLC [34]. Their analysis, which considered testing costs, medical expenses related to testing, and the estimated costs of delaying care, found that NGS was more cost-effective than single-gene testing. NGS not only had the lowest total cost per patient, including costs from delayed care, but also identified the highest proportion of patients with actionable mutations eligible for approved targeted therapies. Additionally, NGS was associated with the shortest time to start appropriate targeted therapy, leading to substantial cost savings from the Canadian public payer’s perspective.

**Figure 3:**
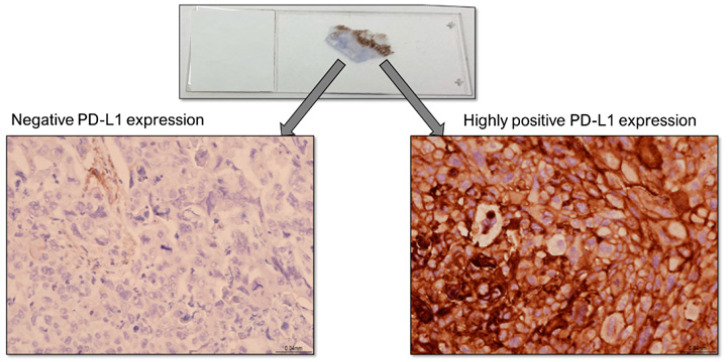
Heterogeneity of PD-L1 expression within small tumor biopsy. Property of Dr. Brendon Sheffield, Department of Laboratory Medicine, William Osler Health System (used with permission from B.S.S.).

When interpreting the results of PD-L1 expression status, one should keep in mind that it is greatly reflective of the small area of biopsy (Figure 3).

When making treatment-related decisions based on PD-L1 expression on pre-operative samples, the threshold for re-testing should be low, especially if the patient’s care could be affected in a significant way.

For patients with no actionable mutations but high PD-L1 expression, the forum favored neoadjuvant ICI + chemotherapy, as supported by neoadjuvant and perioperative trials (Figure 2c). However, if the patient has an EGFR mutation, the forum recommended upfront surgery followed by adjuvant osimertinib, with or without chemotherapy, based on evidence from the ADAURA trial, which demonstrated improved OS with postoperative osimertinib. This recommendation is further supported by the minimal benefit of ICIs in NSCLC harboring EGFR mutations.

In the case of a patient with a RET fusion, there was significant discussion regarding treatment options. Current evidence suggests that patients with RET fusions have minimal benefit from ICIs, and RET fusion testing was not required for the neoadjuvant, adjuvant, or perioperative ICI trials, leaving a gap in understanding their potential inclusion. The benefit of ICIs in early-stage disease for patients with RET fusions and non-KRASG12C actionable mutations remains unclear. In this case, the treatment recommendation includes giving traditional trimodality therapy with neoadjuvant cCRT followed by surgical resection or resection and then considering adjuvant atezolizumab or pembrolizumab after surgery (Figure 2c). The role of the RET fusion inhibitor, selpercatinib, as adjuvant therapy after either surgery or definitive chemoradiation is currently being addressed by the LIBRETTO-432 clinical trial (NCT04819100).

Concerns about administering neoadjuvant ICI + chemotherapy before surgery include the risk of disease progression before the surgical intervention. Neoadjuvant strategies might delay surgery due to treatment-related morbidity or toxicities, potentially leading to surgery cancellations and disease progression. Trials of neoadjuvant or perioperative approaches have shown that up to 20% of patients did not undergo surgery, with 5–10% experiencing disease progression that precluded surgery. A subanalysis of the CheckMate 816 trial [35] found that the majority of patients who did not receive surgery had stage III disease, while only two stage IIA patients were unable to undergo surgery due to distant recurrence. This suggests that neoadjuvant ICI + chemotherapy for stage II NSCLC does not necessarily prevent surgery, and patient selection for patients offered neoadjuvant ICI + chemotherapy for stage III is paramount. Among patients who did not undergo surgery, the median time to death or distant metastases (TTDM) was 24.8 months with nivolumab plus chemotherapy versus 15.6 months with chemotherapy alone (HR, 0.63). Additionally, a similar proportion of patients in both groups received subsequent treatments, including radiotherapy, surgery, and systemic therapy.

Although patients in the ADAURA and ALINA trials did not receive neoadjuvant cCRT, we discussed and agreed that, based on data extrapolation of trimodality approaches, neoadjuvant cCRT could also be considered for patients with activating EGFR or ALK alterations with known N2 involvement found on preoperative staging [36]. Post-surgery, these patients should in addition receive adjuvant osimertinib or alectinib with this approach. The choice of neoadjuvant cCRT vs. chemotherapy + ICI for patients with resectable stage III and other driver mutations needs to take into consideration the benefits of ICI in metastatic settings (i.e., minimal benefit with ICI in patients with RET and ROS1 fusions; patients with smoking history and METexon14 skipping alteration are likely to benefit from ICI). The discussion then needs to be tailored to each patient, considering pCR rates of neoadjuvant cCRT versus offering chemotherapy + ICI. For patients with driver mutations, adjuvant targeted therapy post-surgery is available for EGFR+ (ADAURA trial) and likely will be available for ALK patients (ALINA) trials. The LAURA trial demonstrated the benefits of osimertinib in unresectable stage III EGFR-mutated NSCLC following definitive cCRT, and the ongoing LIBRETTO-432 trial is investigating selpercatinib as an adjuvant therapy after either surgery or cCRT in early stage RET fusion-positive NSCLC (NCT04819100). The results of these trials will likely impact clinical practice in the near future.

The use of neoadjuvant targeted therapy before definitive cCRT for unresectable stage III NSCLC with driver mutations has been explored in a phase 2 proof-of-concept study [37]. In this study, treatment-naïve, non-operable stage III EGFR-mutant NSCLC patients received 80 mg of osimertinib daily for 12 weeks before undergoing definitive radiation therapy and/or surgery. Of the 20 patients enrolled, 13 received radiation, 3 underwent surgery after downstaging (with 1 receiving additional radiation post-surgery), and 5 were excluded. Among the three patients who had surgery, two achieved a pT1aN0 stage, and one achieved a pCR. The median gross tumor volume (GTV), planned tumor volume (PTV), and percentage of total lung volume receiving more than 20 Gy (V20%) significantly decreased after osimertinib treatment, resulting in a lower volume of radiated lung tissue and potentially reduced risk of radiation-related toxicities. As there are reports of increased risk of toxicity when osimertinib is given concurrently with radiation, these therapies should not be given at the same time but rather subsequently. The NeoADAURA clinical trial is investigating this question in a phase III clinical trial (NCT04351555).

## 5. Evaluation of Neoadjuvant Response

The International Association for the Study of Lung Cancer (IASLC) outlined recommendations for processing lung cancer resection specimens and defined pathologic response, including MPR or pCR after neoadjuvant therapy [38]. A standardized approach is to record the type of therapy and assess the percentages of (1) viable tumor cells, (2) necrosis, and (3) stroma (including inflammation and fibrosis), to a total of 100%. This applies to all systemic therapies, including chemotherapy, cCRT, targeted therapy, and immunotherapy. The definition of pCR (0% of residual viable tumor cells) and MPR (<10% of residual viable tumor cells) after neoadjuvant therapy is based on a study conducted in 1997 [39]. In that trial, the achievement of <10% of viable tumor cells post neoadjuvant cCRT resulted in a significantly longer median survival period compared to patients with ≥10% viable tumor cells (27.9 months vs. 13.7 months; *p* = 0.020).

Reporting pathologic responses is challenging and requires additional cost and resources. This is where digital pathology, image analysis, and artificial intelligence tools will help. In the meantime, it is up to individual teams to decide whether the exact percentage of viable tumor cells, if above 10%, is relevant for treatment decisions. It is also crucially important for medical oncologists to flag cases that had neoadjuvant therapy for the pathologist. Pathologists also need to be educated on how to report responses and standardize reporting.

## 6. Conclusions

This report summarizes the outcomes of the Ontario Forum, attended by multi-disciplinary physicians from 23 Ontario Cancer Centres, and highlights current challenges and controversies in the management of early-stage NSCLC that need to be addressed.

Neoadjuvant therapy creates a paradigm shift requiring early referral to medical oncology and encouragement of MCC discussion prior to proceeding with upfront surgical resections. PD-L1 expression, nodal involvement, smoking status, and the presence of actionable genomic alterations are critical factors influencing treatment decisions for early-stage NSCLC. Timely access to preoperative biopsies and molecular testing results is essential for informing treatment decisions and offering patients the best chance of a cure.

Attempts are underway to collect and analyze real-world data on outcomes for early-stage NSCLC patients treated with new approaches. This data will contribute to refining treatment practices. Additionally, criteria for referring patients to MCC are being developed to ensure consistency and efficiency in management. Ongoing efforts aim to streamline the management of early-stage NSCLC, particularly for complex cases such as patients considered borderline resectable, those with unclear margins post-resection, inadequate response to neoadjuvant therapy, or early relapse after treatment. Collaboration among centers is crucial for sharing experiences and insights. Lung cancer-treating physicians in Ontario are planning follow-up meetings and regular updates of this document to ensure continuous improvement in patient care.

## Figures and Tables

**Table 1:** Multicenter international phase 3 trials in NSCLC adjuvant and neoadjuvant settings.

Trial	Enrolment Criteria	Treatment Approach	Outcomes	Relevant Observations
Unresectable Stage 3 NSCLC				
PACIFIC [8,9] (NCT02125461)	Stage III unresectable NSCLC (AJCC seventh edition) with no disease progression following cCRT. No restrictions by the level of PD-L1 expression or the presence of oncogenic driver mutations.	2:1 randomization to durvalumab (10 mg/kg IV) or placebo every 2 weeks for up to 12 months.	Median OS (months): Durvalumab (47.5); Placebo (29.1) [HR 0.72 (95% CI 0.59–0.89)]. Median PFS (months): Durvalumab (16.9); Placebo (5.6) [HR 0.55 (95% CI 0.42-0.65)]. 5-year OS (%): Durvalumab (42.9); Placebo (33.4).	40% of patients with stage IIIA N2 disease: A post hoc exploratory analysis demonstrated similar treatment benefits with durvalumab regardless of stage IIIA N2 status. 43 patients with EGFR or ALK aberrations: A post hoc subgroup analysis in patients with EGFR-mutated tumors showed similar PFS and OS outcomes with durvalumab and placebo (median PFS: 11.2 vs. 10.9 months (HR 0.91); median OS: 46.8 vs. 43.0 months (HR 1.02), with wide confidence intervals).
LAURA [10] (NCT03521154)	Stage III unresectable EGFR (Ex19del/L858R) mutated NSCLC (AJCC eighth edition) with no disease progression following cCRT.	2:1 randomization to osimertinib (80 mg QD) or placebo until objective radiological disease progression.	Median PFS by BICR (months): Osimertinib (39.1 [31.5–NE]); Placebo (5.6 [3.7–7.4]) [HR 0.16 (95% CI 0.10–0.24), *p* < 0.001]. Median OS (months): Osimertinib (54.0 [46.5–NE]); Placebo (NR [42.1–NE]) [HR 0.16 (95% CI 0.10–0.24), *p* = 0.530].	Interim OS data showed a positive trend in favor of osimertinib, despite a high proportion of patients crossing over to osimertinib in the placebo arm (81%).
Resectable NSCLC: Adjuvant Therapy				
ADAURA [11,12] (NCT02511106)	Resected stage IB (including tumors 3-4 cm in size or smaller tumors with visceral pleural invasion) to IIIA NSCLC (AJCC seventh edition) EGFR (Ex19del/L858R) mutated NSCLC. The use of adjuvant chemotherapy was at the discretion of the treating clinician.	1:1 randomization to osimertinib (80 mg once daily) or placebo for 3 years.	4-year DFS Stage IB (%): Osimertinib (80); Placebo (60%) [HR 0.44 (0.25–0.760)]. Stage II (%): Osimertinib (75); Placebo (43) [HR 0.33 (0.21–0.50)]. Stage III (%): Osimertinib (66); Placebo (16) [HR 0.22 (0.15–0.31)]. 5-year OS Overall population (%): Osimertinib (88); Placebo (78) [HR 0.48 (0.34–0.70), *p* < 0.001]. Stage IB (%): Osimertinib (94); Placebo (88) [HR 0.44 (0.17–1.02)] Stage II (%): Osimertinib (85); Placebo (78) [HR 0.63 (0.34–1.12)]. Stage III (%): Osimertinib (85); Placebo (67) [HR 0.37 (0.20–0.64)].	Subanalyses demonstrated that osimertinib reduces the risk of disease recurrence in the CNS (HR 0.24 [CI 0.14–0.42]).
ALINA [13] (NCT03456076)	Resected stage IB (>4 cm)–IIIA ALK rearranged NSCLC (AJCC seventh edition).	1:1 randomization to alectinib (600 mg twice daily) or up to 4 cycles of IV platinum-based chemotherapy.	36-month DFS (%): Alectinib (88.7); Chemotherapy (54.0) [HR 0.24 (95% CI: 0.13–0.43)].	A clinically meaningful CNS-DFS benefit was also observed (HR 0.22 [95% CI 0.08–0.58]).
IMpower010 [14,15] (NCT02486718)	Resected stage IB (>4 cm) to IIIA NSCLC (AJCC seventh edition). Patients with driver mutations were included, and approximately 15% of patients had EGFR/ALK mutations, while 40% had unknown biomarker status. Pneumonectomy was performed in 15%-17% of patients in the trial.	1:1 randomization after adjuvant platinum-based chemotherapy to adjuvant atezolizumab (1200 mg every 21 days; for 16 cycles or 1 year) or best supportive care (observation and regular scans for disease recurrence).	In patients with stage II-IIIA and PD-L1 ≥ 1%, 5-year OS (%): Atezolizumab (76.8); BSC (67.5) [HR 0.71 (95% CI 0.49–1.03)].	In patients without EGFR/ALK mutations and with a PD-L1 score of ≥50%, the difference in OS between atezolizumab and BSC was even more pronounced (5-year OS atezolizumab: 89.1% vs. BSC: 77.5% [HR 0.42 (0.23–0.78)]. This had an impact on the indication and reimbursement approval, which is limited to this subgroup.
PEARLS/KEYNOTE-091 [16] (NCT02504372)	Resected stage IB (>4 cm) to IIIA NSCLC (AJCC seventh edition). Adjuvant chemotherapy was not mandatory but to be considered for stage IB and strongly recommended for stage II and IIIA NSCLC. EGFR mutation and ALK rearrangement status were assessed locally at the discretion of the investigator, and known status was not required for enrolment. Approximately 60% of the enrolled patients had unknown EGFR/ALK status, and 7% of patients had EGFR/ALK mutations (vs. 15% in the IMpower010 trial).	1:1 randomization to pembrolizumab 200 mg or placebo, both administered IV Q3W for up to 18 cycles.	Median DFS (months): Pembrolizumab (53.6) [95% CI 39.2 -NR]; Placebo (42.0) [31.3 -NR], [HR 0.76 (95% CI 0.63–0.91), *p* = 0.0014]. Median OS: Not reached in either group (HR 0.87 [95% CI 0.67–1.15]; *p* = 0.17). 3-year OS (%): Pembrolizumab (82); Placebo (80).	Approximately 80% of participants received 3–4 cycles, and 6% received 1–2 cycles of previous adjuvant chemotherapy with a cisplatin- or carboplatin-based regimen, or both. Subgroup analyses indicated that patients receiving adjuvant chemotherapy, even 1–2 cycles, had the greatest benefit from adjuvant pembrolizumab (HR 0.73 [95% CI 0.60–0.89]) compared to those who did not receive adjuvant chemotherapy (HR 1.25 [95% CI 0.76–2.05]. This had impact on the indication by Health Canada that patients must have at least one cycle of adjuvant chemotherapy.
Resectable NSCLC: Neoadjuvant ICI Strategy				
CheckMate 816 [17] (NCT02998528)	Resectable stage IB-IIIA NSCLC (AJCC seventh edition), EGFR/ALK wildtype. Surgery was planned within 6 weeks of the completion of neoadjuvant treatment, after which patients in both groups could receive up to four cycles of adjuvant chemotherapy, radiation therapy, or both.	1:1 randomization to 3 cycles of neoadjuvant nivolumab + carboplatin/paclitaxel or carboplatin/paclitaxel.	pCR (%): Nivolumab + carboplatin/paclitaxel (24); Carboplatin/paclitaxel (2.2) [*p* < 0.001]. Median EFS (months): Nivolumab + carboplatin/paclitaxel (31.6); Carboplatin/paclitaxel (20.8) [HR 0.63 (97.38% CI 0.43–0.91), *p* = 0.005] 3-year EFS (%): Nivolumab + carboplatin/paclitaxel (57); Carboplatin/paclitaxel (43) 3-year OS (%): Nivolumab + carboplatin/paclitaxel (78); Carboplatin/paclitaxel (64).	The magnitude of EFS benefit was greater in patients with stage IIIA disease, PD-L1 expression ≥ 1%, and/or non-squamous histology. Surgery: Nivolumab+ chemotherapy: 83.2% (16.8% pneumonectomy). Chemotherapy: 75.4% (16.8% pneumonectomy). Surgical outcomes: Without R0 resection: 14.5% Patients without surgery or R0 resection: 29.1% R0 rate: 83% Grade 3–4 AEs: 11.4% 90-day Mortality: 3.4%
Resectable Stage IB-III NSCLC: Perioperative ICI Strategies				
KEYNOTE-671 [18] (NCT03425643)	Stage II–IIIB NSCLC (AJCC eighth edition, EGFR/ALK wildtype.	1:1 randomization to pembrolizumab (200 mg) or placebo every 3 weeks, both in combination with platinum-doublet chemotherapy for 4 cycles followed by adjuvant pembrolizumab (200 mg) or placebo every 3 weeks for a maximum of 13 cycles.	MPR (%): Pembrolizumab (30.2); Placebo (11.0) [*p* = 0.000]. pCR (%): Pembrolizumab (18.1); Placebo (4.0) [*p* < 0.0001]. After a median follow-up of 36.6 months, median OS (months): Pembrolizumab (NR, 95% CI NR-NR); Placebo (52.4, 95% CI 45.7–NR) [HR 0.72 (95% CI 0.56–0.93), *p* = 0.00517]. 36-mo OS rates (%): Pembrolizumab (71.3); Placebo (64.0).	Surgery: Pembrolizumab: 82% Placebo: 79% (the percentage of pneumonectomies was similar in the two arms [12%]. Surgical outcomes: Without R0 resection: 8% Without surgery or R0 resection: 25% R0 rate: 92% Grade 3–4 AEs: 23% 90-day mortality: 4% 290 (73.2%) patients received ≥1 dose of adjuvant pembrolizumab. The relative benefit in the pembrolizumab group increased with increasing PD-L1 expression HR for EFS: PD-L1 ≥ 50%: 0.42 (0.28–0.65) PD-L1 1 to 49%: 0.51 (0.34–0.75) PD-L1 < 1%: 0.77 (0.55–1.07)
CheckMate 77T [19] (NCT04025879)	Resectable stage IIA (>4 cm)–IIIB (N2) NSCLC (AJCC eighth edition), EGFR/ALK wild type.	1:1 randomization to nivolumab (360 mg) or placebo every 3 weeks, both in combination with platinum-doublet chemotherapy for 4 cycles, followed by adjuvant nivolumab (480 mg) or placebo every 4 weeks for 1 year vs. neoadjuvant chemotherapy	Median EFS (months): Nivolumab (NR; 28.9–NR); Placebo (18.4; 13.6–28.1) [HR 0.58 (97.36% CI 0.42–0.81; *p* < 0.0001]. pCR (%): Nivolumab (25.3); Placebo (4.7) [OR 6.64 (95% CI, 3.40–12.97)]. MPR (%): Nivolumab (35.4); Placebo (12.1) [OR 4.01 (2.48–6.49)].	Surgery: Nivolumab: 78% (9% pneumonectomy) Placebo: 77% (9% pneumonectomy) R0 rate: 89% Surgery-related AEs: 12% The nivolumab benefit was more pronounced in patients with stage III disease, those with squamous histology, and those whose tumors expressed PD-L1 at ≥50%. HR for EFS: PD-L1 ≥ 50%: 0.26 (0.12–0.55) PD-L1 1 to 49%: 0.76 (0.46–1.25) PD-L1 < 1%: 0.73 (0.47–1.15)
AEGEAN [20] (NCT03800134)	Resectable stage IIA–IIIB (cN2, mediastinal lymph node involvement) NSCLC (AJCC eighth edition).	1:1 randomization to durvalumab (1500 mg) or placebo once every 3 weeks, both in combination with platinum doublet chemotherapy for 4 cycles, followed by adjuvant durvalumab (1500 mg) or placebo every 4 weeks for 12 cycles	pCR (%): Durvalumab (17.2); Chemotherapy (4.3) [*p* = 0.00004]. Median EFS (months): Durvalumab (NR); Chemotherapy (25.9) [HR 0.68 (95% CI 0.53–0.88), *p* = 0.004].	Surgery: Durvalumab: 77.6% (7.6% pneumonectomy) Placebo: 76.7% (7.8% pneumonectomy) Surgical outcomes: Without R0 resection: 8.8% Patients without surgery or R0 resection: 26.5% R0 rate: 96% The relative benefit in the durvalumab group increased with increasing PD-L1 expression. HR for EFS PD-L1 ≥ 50%: 0.60 (0.35–1.01) PD-L1 1 to 49%: 0.70 (0.46–1.05) PD-L1 < 1%: 0.76 (0.49–1.17)
Neotorch [21] (NCT04158440)	Patients scheduled for pneumonectomy and those with T4 disease (defined by any criterion other than tumor diameter ≥ 7 cm) were excluded.	1:1 randomization to toripalimab (240 mg) or placebo once every 3 weeks, both in combination with platinum-based chemotherapy for 3 cycles before surgery and 1 cycle after surgery, followed by toripalimab only (240 mg) or placebo once every 3 weeks for up to 13 cycles.	Only patients with stage III disease included in the interim analysis. Median EFS (months): Toripalimab (NR; 95% CI, 24.4 months-NR); Placebo (15.1; 95% CI, 10.6-21.9) [HR 0.40 (95% CI 0.28–0.57), *p* < 0.001]. MPR (%): Toripalimab (48.5; 95% CI, 41.4–55.6%); Placebo (8.4; 95% CI 5.0–13.1%). Between group difference (40.2; 95% CI, 32.2–48.1%, *p* < 0.001). pCR (%): Toripalimab: 24.8% (95% CI 19.0–31.3%) Placebo: 1.0% (95% CI 0.1%-3.5%) (between group difference, 23.7% [95% CI, 17.6–29.8%]).	Surgery in stage III patients: Toripalimab: 82.2% (166/202; 9% pneumonectomy) Placebo: 73.3% (148/202; 9.5% pneumonectomy) Adjuvant therapy: Toripalimab: 71.3% (144/202) Placebo: 64.9% (131/202) The relative benefit with toripalimab increased with increasing PD-L1 expression HR for EFS (stage III disease) PD-L1 ≥ 50%: 0.31 (0.15–0.60) PD-L1 1 to 49%: 0.31 (0.17–1.54) PD-L1 < 1%: 0.65 (0.33–1.23)
RATIONALE-315 [22] (NCT04379635)	Resectable stage II or III NSCLC (AJCC eighth edition), EGFR/ALK wild type.	1:1 randomization to 3–4 cycles of neoadjuvant tislelizumab (200 mg) or placebo (IV Q3W) plus chemotherapy, then surgery and ≤8 cycles of adjuvant tislelizumab (400 mg) or placebo (IV Q6W).	Median study follow-up: 22.0 months (range: 0.1–38.4) EFS (%): Tislelizumab (68.3); Placebo (36.6) [HR, 0.56 (95% CI, 0.40–0.79), *p* = 0.0003]. OS (%): Tislelizumab (88.6); Placebo (79.4) [HR, 0.62 (95% CI, 0.39–0.98), *p* = 0.0193]. MPR (%) Tislelizumab (56.2); Placebo (15). Between group difference (41.1; 95% CI: 33.2, 49.1, *p* < 0.0001). pCR (%) Tislelizumab (40.7); Placebo (5.7%). Between group difference (35; 95% CI: 27.9, 42.1, *p* < 0.0001).	Surgery: Tislelizumab: 190 (84.1%); Placebo: 173 (76.2%). Adjuvant therapy: Tislelizumab: 168 (74.3%); Placebo: 147 (64.8%). HR for EFS: PD-L1 ≥ 50%: 0.71 (0.38, 1.34) PD-L1 1 to 49%: 0.34 (0.17, 0.66) PD-L1 < 1%: 0.80 (0.47, 1.38)

AEs, adverse events; AJCC, American Joint Committee on Cancer; ALK, anaplastic lymphoma kinase; BICR, blinded independent central review; BSC, best supportive care; cCRT, concurrent chemoradiation therapy; CI, confidence interval; cN2, clinical mediastinal involvement; CNS, central nervous system; DFS, disease-free survival; EFS, event-free survival; EGFR, epidermal growth factor receptor; HR, hazard ratio; IV, intravenous; MPR, major pathologic response; N2, ipsilateral and/or subcarinal mediastinal lymphatic spread; NSCLC, non-small cell lung cancer; OR, odds ratio; OS, overall survival; pCR, pathologic complete response; PD-L1, programmed death ligand 1; PFS, progression-free survival; Q3W, once every 3 weeks; Q6W, once every 6 weeks; R0, no residual tumor.

**Table 2:** Pre-meeting survey: Current treatment patterns for early-stage NSCLC in Ontario.

Stage	Treatment Approaches/Considerations
Stage IA (T1aN0, T1bN0, T1cN0; LN < 3 cm) or Stage IB (T2aN0; nodes > 3 cm but <4 cm)	Surgery and SBRT are standards of care in addition to clinical trials. Other ablative modalities or palliative radiation are considered in patients who are not surgical candidates.
IIA (T2bN0; nodes > 4 cm but <5 cm)	As PD-L1 expression increases, the use of neoadjuvant and adjuvant ICI increases. No difference by histology. Smoking impact decisions in patients with PD-L1 expression is 0%.
IIB NSCLC (both node positive and negative populations)	Increased use of systemic therapy Increased use of ICI by PD-L1 expression status Increased preference for neoadjuvant approach No difference in preference by histology Smoking impacted decisions if PD-L1 expression is 0%
IIIA T3/T4 with N1	Preferred options in the following order: Neoadjuvant chemotherapy + ICI Perioperative chemotherapy + ICI PACIFIC trial protocol (durvalumab after cCRT)
IIIA T1/T2 with N2	Histology does not impact preference for treatment approaches PD-L1 status and smoking impact treatment preferences Preference for the PACIFIC trial (durvalumab after cCRT) protocol increased by nodal involvement (bulky and multimodal stations)
IIIB T3N2 or IIIB T4N2	The PACIFIC trial protocol (durvalumab after cCRT)
If EGFR/ALK/ROS1 genetic alterations	In general, ICI is not considered

ALK, anaplastic lymphoma kinase; cCRT, concurrent chemoradiation therapy; EGFR, epidermal growth factor receptor; ICI, immune checkpoint inhibitor; PD-L1, programmed death ligand 1; ROS1, c-ros oncogene; SBRT, stereotactic body radiotherapy.

**Table 3:** Operability/resectability criteria.

Patient wishes and risk tolerance
2. Surgeon experience and risk tolerance No surgeons should be pushed to perform an operation they do not feel comfortable performing and without the environment and resources needed for good outcomes
3. Baseline physiology (PFTs, VO_2_ max, 6-min walk test, quantitative V/Q)
4. ECOG PS, nutrition status, overall exercise tolerance, lifestyle, and comorbidities
5. Feasibility of R0 at baseline
6. Feasibility of R0 based on expected response or risk of progression by neoadjuvant approach (guided by biomarker profile, patient characteristics, functional reserve, type of neoadjuvant regimen employed, surgical experience)
7. Predicted post-operative functional reserve/QoL based on the extent of pulmonary resection required for R0

PFTs, pulmonary function tests; VO_2_ max, maximum rate of oxygen consumption attainable during physical exertion; V/Q, ventilation/perfusion scan; ECOG PS, Eastern Cooperative Oncology Group performance status; R0, no residual tumor; QoL, quality of life.

**Table 4:** Key takeaways on resectability and the impact of nodal disease.

Resectability	The decision regarding resectability is still made upfront prior to neoadjuvant therapy, as there is no data that any new approach could make unresectable tumors resectable. Patients considered “borderline” resectable were not included in the neoadjuvant/perioperative trials Resectability is defined by the surgical team based on their assessment of whether “it can be all taken out, with negative resection margins, without compromising patient’s quality of life”. In most centers across Ontario, treatment decisions regarding preoperative approaches and resectability are made in collaboration at a multidisciplinary cancer conference. ○ When deciding whether to offer neoadjuvant chemotherapy + ICI, disease-(PD-L1 expression, presence of driver mutations, number of positive nodes, disease burden, etc.) and patient-related characteristics (smoking status, comorbidities, autoimmune conditions, performance status, age/frailness, etc.) are taken into consideration.
Bulky vs. non-bulky nodal disease	According to the guidelines, non-bulky lymph nodes are usually described as lymph nodes with a diameter of <3 cm, easily measurable and free of major mediastinal structures, including the trachea and great vessels, or low-volume lymph nodes [25,26,27,28].

ICI, immune checkpoint inhibitor; PD-L1, programmed death ligand 1.
